# Multi-Criteria Optimization of Cost-Effective and Environmentally Friendly Reactive Powder Concrete Incorporating Waste Glass and Micro Calcium Carbonate

**DOI:** 10.3390/ma16196434

**Published:** 2023-09-27

**Authors:** Joaquín Abellán-García, Nemesio Daza, Marielena Molinares, Yassir M. Abbas, Mohammad Iqbal Khan

**Affiliations:** 1 Department of Civil and Environmental Engineering, Universidad Del Norte, Barranquilla 081007, Colombia; 2 Department of Civil Engineering, Universidad Simón Bolivar, Barranquilla 111321, Colombia; nemesio.daza@unisimon.edu.co; 3 Department of Industrial, Manufacturing, and Systems Engineering, Texas Tech University, Lubbock, TX 79409, USA; marielen@ttu.edu; 4 Department of Civil Engineering, College of Engineering, King Saud University, P.O. Box 800, Riyadh 11421, Saudi Arabia; yabbas@ksu.edu.sa

**Keywords:** RPC, multicriteria optimization, particle packing density, waste glass powder

## Abstract

In pursuit of developing an eco-friendly and cost-effective reactive powder concrete (RPC), we utilized a multi-objective optimization technique. This approach pivoted on the incorporation of byproducts, with a spotlight on ground glass powder (GP) as a pivotal supplementary cementitious material (SCM). Our goal was twofold: engineering cost-efficient concrete while maintaining environmental integrity. The derived RPC showcased robust mechanical strength and impressive workability. Rigorous evaluations, containing attributes like compressive strength, resistance to chloride ion penetration, ultrasonic pulse speed, and drying shrinkage, highlighted its merits. Notably, the optimized RPC, despite an insignificant decrease in compressive strength at 90 days compared to its traditional counterpart, maintained steady strength augmentation over time. The refinement process culminated in a notable 29% reduction in ordinary Portland cement (OPC) usage and a significant 64% decrease in silica fume (SF), with the optimized mix composition being 590 for cement, 100 for SF, 335 for GP, and 257 kg/m^3^ for calcium carbonate. Additionally, the optimized RPC stood out due to the enhanced rheological behavior, influenced by the lubricative properties of calcium carbonate and the water conservation features of the glass powder. The reactive properties of SF, combined with GP, brought distinct performance variations, most evident at 28 days. Yet, both mixtures exhibited superior resistance to chloride, deeming them ideal for rigorous settings like coastal regions. Significantly, the RPC iteration, enriched with selective mineral admixtures, displayed a reduced tendency for drying-induced shrinkage, mitigating potential crack emergence.

## 1. Introduction

The increase in greenhouse gas emissions from ordinary Portland cement (OPC) has led to a growing demand for sustainable cementitious materials. Cement production is responsible for more than 8% of the world’s carbon dioxide emissions, making it crucial to reduce its environmental impact [1,2]. Developed in the 1990s for the building industry, reactive powder concrete (RPC) is an innovative type of cement composite with exceptional strength and superior characteristics compared to conventional concrete [3]. RPC’s microstructure features a high density of fine, and mostly reactive, particles, which leads to low porosity and high compressive strength (CS). It also exhibits high tensile strength and ductility, making it capable of withstanding high stress and deformation without cracking or fracturing [4].

Although RPC has been widely recognized as a superior material for construction, its production has been limited in many countries, particularly in developing nations, due to several factors. As noted by Peng et al. [5] and Mayhoub [6], the hard-to-find aspect and high cost of some of the component elements, such as high-performance silica fume and ultrafine powders, make it difficult to produce RPC at a large scale, especially in areas where these materials are not readily available [1,2]. Furthermore, the use of these materials can also result in environmental degradation and unsustainable resource consumption. As such, researchers have been exploring alternative methods for RPC production, such as using waste materials or locally sourced resources, in order to make RPC more accessible and sustainable [7,8]. According to Nasr et al. [9], incorporating waste materials such as fly ash and slag can reduce the environmental impact of RPC and lower its production costs while maintaining its superior properties. Such efforts are crucial for making RPC more widely available and sustainable, particularly in regions where traditional cement-based materials are not ideal due to their lower durability and strength.

Further from the carbon footprint problems, the increased cement need in RPC has various negative implications on the performance of the concrete due to shrinkage and microcracking concerns [3]. Furthermore, specific and more expensive ingredients such as silica fume (SF) play an important role in RPC, improving the microstructure of this particular concrete [4,10]. The addition of SF to RPC can enhance its mechanical qualities [4,11] (and durability) by decreasing its permeability to water and chemicals and enhancing its resilience to freeze–thaw cycles [7,12,13].

Several researchers have conducted experimental works to analyze the effect of SF dosage on the properties of reactive powder concrete (RPC) [14]. According to Al-Hassani et al. [15], increasing the SF content in RPC resulted in a significant increase in compressive strength but a comparatively modest improvement in tensile strength. Additionally, the addition of steel fibers improved the tensile strength and load-deflection behavior of RPC. Chan and Chu [14] discovered that incorporating SF significantly increased the bond strength between fiber and matrix, particularly the fiber pullout energy. The authors state that for bond properties, the ideal SF dose ranged between 20 and 30% [11,14]. However, the high cost and influence of SF on the water-to-binder ratio, and thus on concrete shrinkage, are some of the drawbacks associated with this high-silicon-oxide and ultra-fine mineral admixture [16,17].

On the other hand, as recycled glass (GP) is used progressively more, the capacity of landfills to properly handle it and dispose of it permanently is being exceeded [18]. It is discouraging that while being a highly recyclable material, it leaves behind a significant amount of waste when disposed of in quarries due to the fact that it is not biodegradable [19]. While this problem remains, interest in integrating GP as a study material for implementation within the construction industry, particularly in the development of structural elements [20], has increased due to its accessibility, low cost, and favorable properties within the mixtures [21,22]. In turn, this helps lessen its environmental impact [23].

Numerous studies in the sector have recently focused on recycled glass as a potential concrete component to enhance its qualities, affordability, and carbon footprint. According to Kaminsky et al. [24], the properties of the treated GP allow for the possibility of using it as a supplementary cementitious material (SMC) in concrete mixtures. A study by Sadiqul et al. [23] looked into using GP as a binder in mortar and concrete mixtures when combined with cement. According to these researchers, they increased compressive strength by 8% above the control mixture by employing 20% GP as a binder.

Du and Tan [25], who examined the mechanical and durability characteristics of concrete mixtures employing GP as a replacement for cement, came to similar conclusions. These authors claimed that when GP content was increased to at least 30%, compressive strength improved by up to 27%, water absorption capacity decreased by 75%, and electrical conductivity decreased by 88%. Similar results were reported by other authors [19,26]. Low Ca/Si ratios are thought to be responsible for these improvements because they induce the production of C-S-H gel, which enhances the microstructure of the cementitious matrix and lowers ion concentrations in the pore solution [25,26]. In a thorough investigation of the mechanical and durability attributes of concrete, Lal Jain et al. [27] substituted varying amounts of GP (5, 10, 15, 20, and 25%) and granite powder (GrP, 10, 20, 30, 40, and 50%) for cement and fine aggregate, respectively. A higher GP dosage led to a rise in compressive strength (CS) at 28 days compared to the control mix, and mixtures containing 20% GP and 0% GrP showed a significant 24.8% increase in strength. Furthermore, concretes containing 15% GP and 30% GrP revealed a 34% increase in CS over the control combination. Kou and Xing [22] achieved similar results when they explored the use of recycled GP (15% and 30% by weight) in combination with SF as SMCs for fiber-reinforced UHPC mixtures. According to their findings, larger GP dosages resulted in decreased workability, while CS increased by 7.0% and 2.8%, respectively, when compared to the control mixture. The chemical stimulation given by GP, which combines with CH to form a low basicity C-S-H, can be linked to the achievement of CS by means of the pozzolanic reaction [21,26].

Al-Awabdeh et al. [28] used a water-to-cement ratio of 0.46 and partially substituted fine and coarse aggregates at levels of 30% and 50% to study the effects of silane-treated glass cullet on concrete mixtures. Glass powder was added to the mixes in amounts ranging from 2% to 5% by weight. The results of this investigation showed that the absorption capacity was significantly reduced by up to 87% when treated broken glass was added. However, compressive strength was reduced by as much as 46% when treated glass was employed to partially replace fine and coarse stones. Castro and Brito [29] studied the use of GP as a replacement for 5, 10, and 20% of fine aggregate in concrete mixes and found that similar results were obtained. The employment of 20% GP boosts concrete workability, according to the researchers. Wang [30] also found similar outcomes when he looked at the impact of using GP as a fine aggregate replacement in three different concrete mix designs with target strengths of 21, 28, and 35 MPa. According to Wang’s study, adding more GP than 20% had a negative impact on how well concrete mixtures worked. In addition, the cementitious matrix’s compressive strength was raised, and chloride ion penetration was decreased when 20% of the fine aggregate was replaced with GP [31,32].

Due to the alkali nature of glass and its high silica content, which is primarily in the amorphous condition, the alkali–silica reaction (ASR) is an expected but adverse behavior that must be properly handled when adding glass waste into the manufacture of concrete. ASR is mostly detected in the later phases of curing as a formation of cracks and expansion of the cement matrix, which ultimately can cause considerable structural damage [33]. However, glass powder silica oxide can react with CH to generate C-S-H when water is included [26], and therefore, optimal dosages and treatments of GP could improve the mechanical properties of concrete compositions. In glass powder concrete, Guo et al. [34] discovered that including (SCMs) such as fly ash can significantly reduce the rate of early-age alkali–silica reaction (ASR) growth while Dhir et al. [35] established that the addition of slag and metakaolin mitigates this expansion. Glass particle reactivity is regulated by size, and particles smaller than 600 m do not generate deleterious ASR even when ASR suppressants are used [36]. These data imply that the negative impacts of using recycled glass in concrete, particularly with regard to ASR, can be mitigated by the use of SCMs or GP particles smaller than the #30 sieve size.

On the other hand, the mechanical properties of ultra-high-strength concrete with particle packing density, such as RPC, may benefit from GP, according to some research. For example, in order to partially substitute cement in RPC, Hussain et al. [37] used GP, and the results showed that adding 20% sustainable GP to cement enhanced CS and flexural behavior. Similar findings were reported by other investigations [38,39].

Furthermore, multiple research projects have been conducted to explore the impact and synergistic effects of various SCMs, in addition to SF, in the construction of environmentally friendly RPC concrete [38,40,41]. Based on the foregoing findings, it is reasonable to conclude that GP offers the potential as a viable option for traditional materials in the development of RPC [42,43].

Despite its benefits, RPC has two key drawbacks: a high cost and a large carbon impact. Numerous research has looked at the use of inexpensive, ecologically benign, and locally accessible minerals as additives in RPC combinations [20,21], but their effects on parameters other than compressive strength (CS) have received little attention. In order to fill this knowledge gap, our project experimentally evaluates a wide variety of mechanical and durability parameters. To serve as binding components in RPC combinations, calcium carbonate and ground glass powders must be combined in the most advantageous, economical, and environmentally responsible way possible.

Numerous endeavors have been made to refine RPC formulations, yet the current research uniquely pivots towards the incorporation of eco-friendly components like waste glass and micro calcium carbonate. Hence, we not only advocate for pioneering waste management solutions but also underscore the significance of sustainable construction methodologies. Beyond formulation, we have explored the performance metrics of these novel RPC mixtures, spanning from their workability to their durability under various conditions, such as chloride ion exposure. The utilized methodology, underscored by the adoption of the Modified Andreasen and Andersen (MAA) model, offers a robust approach to assessing particle distribution and density, amplifying the reliability of the outcomes.

## 2. Materials and Methodology

**Figure 1:**
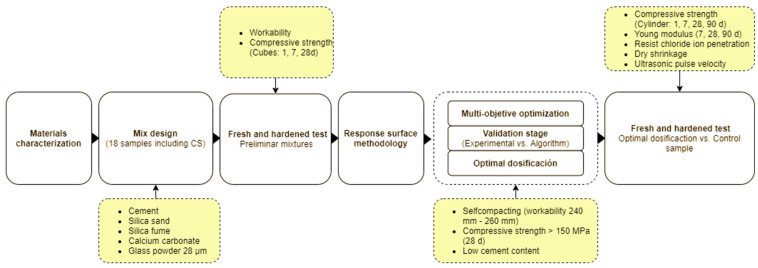
Experimental framework methodology.

Figure 1 presents the testing plan, along with the components used to formulate the RPC, the amounts of the concrete mixture, and the experimental techniques utilized in this study.

### 2.1. Characterization of the RPC-Making Materials

**Figure 2:**
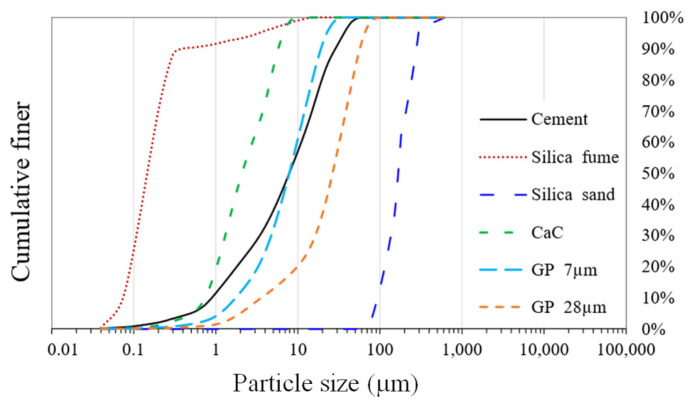
Particle size distribution of granular RPC-making ingredients.

**Figure 3:**
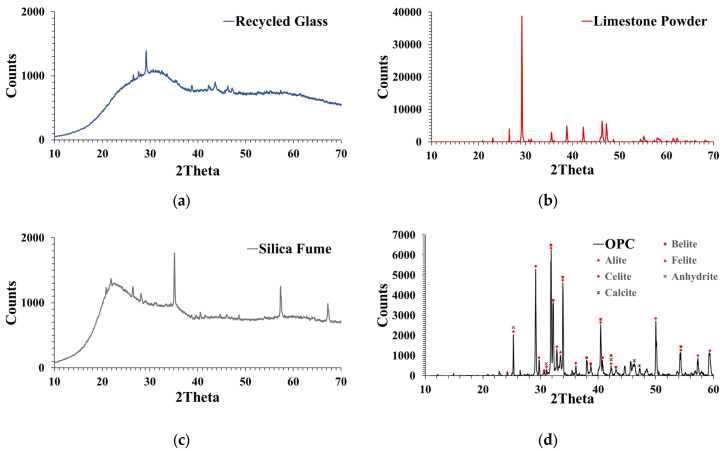
XRD findings on (**a**) waste glass, (**b**) CaC, (**c**) SF, and (**d**) cement.

**Figure 4:**
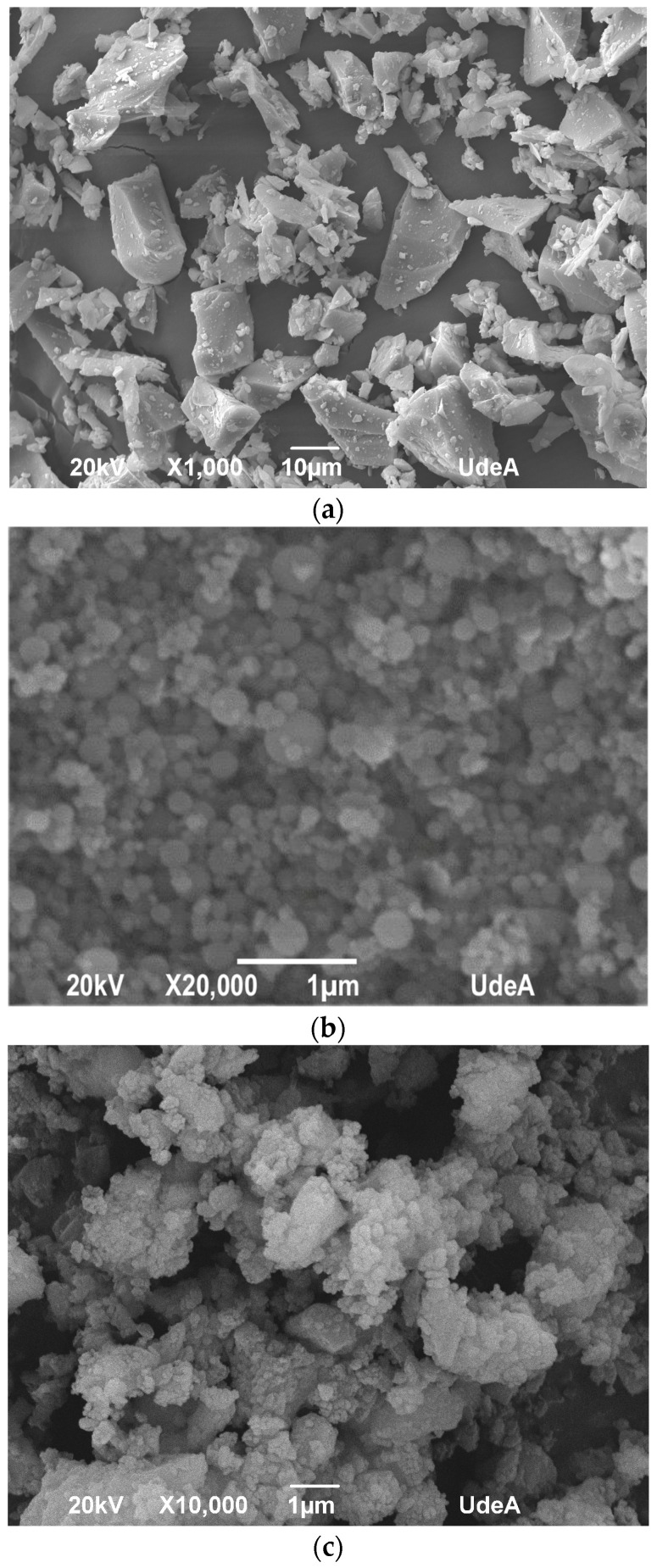

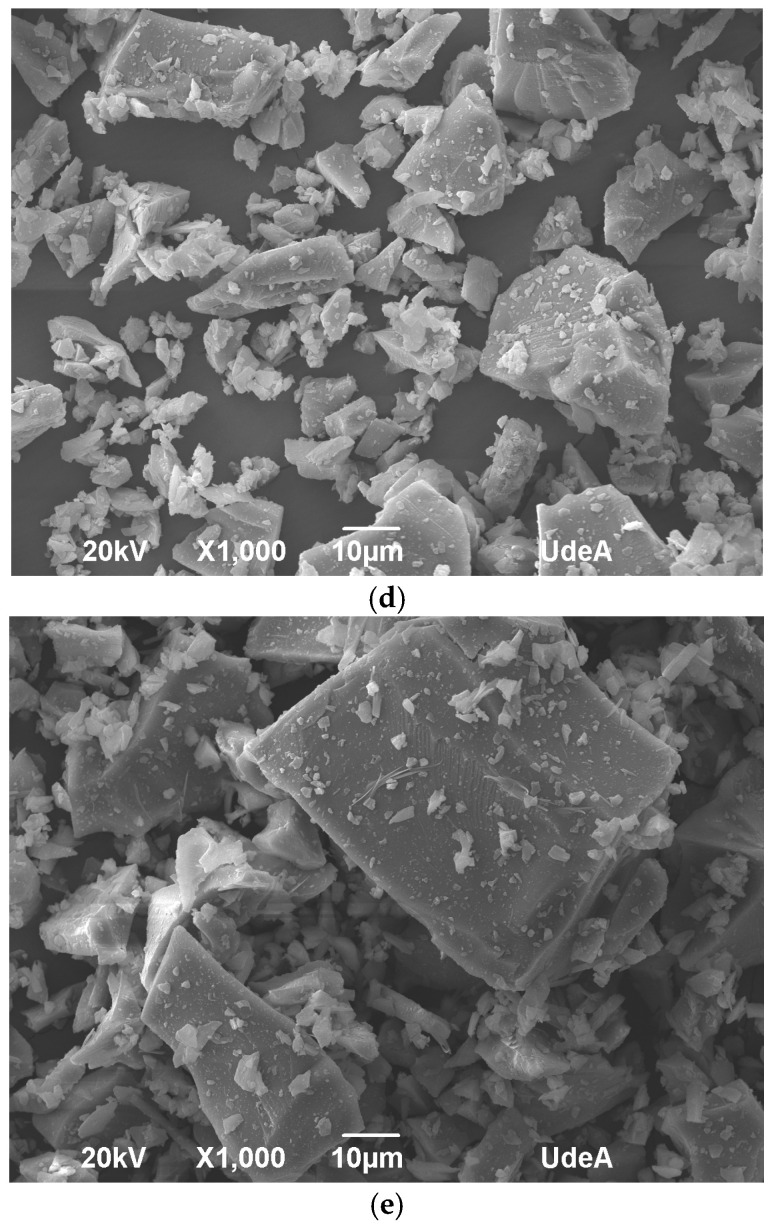
SEM findings of the fine powders employed in this study: (**a**) OPC, (**b**) SF, (**c**) CaC, (**d**) GP 7, and (**e**) GP 28.

The RPC doses were made using raw components from the Colombian market. The cement had a mean particle size of 9 μm and a specific density of 3.14. The employed SF conformed to ASTM C-1240 [44] and had a specific density of 2.21 and a d_50_ of 0.15 μm. Additionally, silica sand was used, which has a d_50_ of 165 m, a maximum particle size of 600 μm (d_max_), and a specific density of 2.65. In order to lower the cement and SF content, calcium carbonate powder (CaC) and waste glass powder (GP) were added to the optimal dose. CaC has a d_50_ value of 2 μm and a specific density of 2.74; the d_50_ value for glass powder was 28 μm. The specific density of both of the glass powders was 2.61. The results of particle size distribution (PSD), the mineralogical investigations carried out using X-ray diffraction (XRD), and the scanning electron microscopy (SEM) study of the constituents are shown in Figure 2, Figure 3 and Figure 4, respectively.

Table 1 and Table 2 summarize the elemental chemical composition obtained by XRF and the physical properties of cement, SF, CaC, and GP28, respectively.

The superplasticizer employed in this study was a high-range water reducer admixture (HRWR) based on polycarboxylate ether. It had a solids concentration of 40% and a specific gravity of 1.07.

### 2.2. Analytical Methodology

#### 2.2.1. Packing Density Model

The packing density of the granular ingredients, especially powders, is crucial for designing an efficient RPC combination [6,45]. The packing density model for RPC design is based on the hypothesis that the mechanical properties of the final concrete may be significantly influenced by the packing density of the powders [40]. The particle size distribution of the each RPC component (PSD) is taken into account by the model to establish the ideal packing density for the mixture. Maximizing the density of the particles in the mixture is the aim of the packing density model [6,46]. This may be conducted by choosing the component’s particle sizes carefully (including powders), as well as by adding the right chemical admixtures to the combination to lower it. A RPC mixture’s packing density may be changed to increase compressive strength and durability, and the inclusion of fibers improves RPC’s post-cracking behavior under flexural or tensile loadings [7,12,47]. Modified Andreasen and Andersen (MAA), a continuous particle packing theory, is used in this study. References [48,49,50] provide further details regarding this particle packing model.

#### 2.2.2. Design and Analysis of Experiments

The design of the experiment is a key component of statistical analysis that seeks to identify relevant factors and their impact on various attributes. The purpose of this research is to provide insight into the changes in RPC properties when SCMs are added to a conventional concrete mixture. The goal is to conduct as few tests as possible while keeping detailed information on the factors, their interactions, and their impact on the responses. An appropriate experimental zone is identified using response surface methodology (RSM).

**Figure 5:**
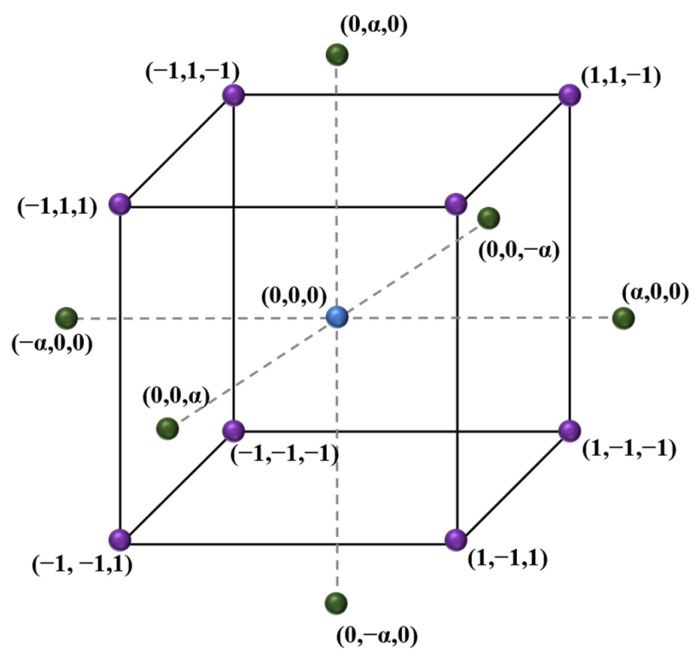
Diagram of a central composite design.

Various methods for data collection can be examined using response surface methodology (RSM), in this case, central composite design (CCD) was selected for the mixing design since it stands out as the most efficient and commonly used in experimental studies. Its advantages include rotatability, exact estimations in all directions, and the ability to generate predictions that lie within the experimental range [51,52,53]. It is required to analyze the connection between variables, ensure the normal behavior and variance of the variables, and validate these assumptions at a 95% confidence level as part of the validation process [54]. Figure 5 represents the central composite design, which depicts the use of center, factorial, and axial design points in the current investigation.

Following previous works [55], the value reflecting the distance of the axial points from the center is fixed at 1.789. Table 3 displays the design point values as well as the associated coordinates in the CCD.

This study takes four repetitions of the central point into account. The design involves three factors: the amount of OPC (ordinary Portland cement) in kg/m^3^ [A], the water-to-binder ratio [B], and the volume ratio of HRWR (high-range water reducer) [C]. The MAA hypothesis is used to determine the remaining RPC component proportions. Table 4 shows the factor values for each design coordinate.

For the final analysis, a multi-objective optimization model is suggested in which concrete features such as self-compacting behavior, compressive strength, and low cement content are treated as response variables, and significant parameters are manipulated as input variables. This sort of optimization employs an analytical method based on the desirability function introduced by Derringer and Suich [56], which is based on a hierarchical model such as the RSM. The usefulness of functions is defined by whether the response must be maximized, minimized, or kept within a specific range, denoted by the upper (U) and lower (L) bounds. The goals intended to be reached using the optimization are specified in Table 5.

The abovementioned criteria optimization allows for the determination of proposed input variable values that can satisfy the desired conditions in response variables. These determined positions, known as simultaneous optimal points, may or may not always coincide with the control parameters. They are, instead, an achievable option that keeps all related variables within acceptable limits [53].

### 2.3. Experimental Methodology

#### 2.3.1. Mixing Procedure and Curing

To begin the mixing process, the HRWR and water were combined and mixed at a minimum speed for 90 s. Afterward, the previously mixed binders were added to ensure homogeneity during mixing. The blending process continued until the mixture achieved a flowing consistency, which typically took 3–5 min, depending on the RPC mixture. Once the desired fluidity was achieved, the mixture was mixed at medium speed for 60 s. Next, the equipment was stopped, and the mold was scraped, after which the mixing process resumed for 180 s at high speed. Silica sand was then added and mixed for 60 s at a slow speed before the mixing process was completed at medium speed for an additional 4 min. The workability of each mixture was evaluated using the guidelines outlined in ASTM C1437 [57].

After the workability test, 50 mm mortar specimens were prepared in accordance with ASTM C109 [58], while concrete cylinders were prepared in accordance with ASTM C39 [59] (experimental phase 2). The molds were vibrated during the concrete pouring process and for an extra 60 s after pouring was finished in order to improve the true packing density of the RPC; to prevent water evaporation, protective paper was placed over both types of specimens. After 24 h, the specimens were demolded and allowed to cure in water at 23 °C according to the ASTM C109 [58] and ASTM C511 [60].

#### 2.3.2. Fresh and Hardened Test

In this study, fresh and hardened concrete characteristics were used to assess the quality of sustainable concrete. Three samples were tested for each test, and the results were averaged. The workability of each mixture was evaluated using the guidelines outlined in ASTM C1437 [57]. For experimental stage 1, concrete cube compression tests were performed at 1, 7, and 28 days in line with ASTM C109 [58]. For experimental stage 2, cylinder compression tests were performed at 1, 7, 28, and 90 days in accordance with ASTM C39 [59]. A chloride resistance test was performed on three concrete discs, each corresponding to a different measurement age (7, 28, and 90 days), in accordance with the procedures outlined in ASTM C1202 [61]. The test involved applying a voltage potential to the discs while they were immersed in a sodium chloride solution.

A drying shrinkage test was conducted on three samples for each mixture according to the guidelines of ASTM C490 [62]. The temperature of the molding room was 23 °C with a relative humidity greater than 50%. Up to the age of 25 days, the sample length is measured and recorded every day. The drying shrinkage is calculated using the length difference between the initial record measurement and the following measurements. An ultrasonic pulse velocity test was carried out according to the guidelines of ASTM C597 [63]. This test determines the quality of concrete by measuring the speed of an ultrasonic pulse through the substance. The test involves a powerful electrical pulse from the transmitter causing the specimen to vibrate, with the receiver positioned on the opposite side. The concrete transmits the vibration to the receiver, and the speed of the wave can be approximated using the specimen length. This technique assesses tangible quality factors such as elastic modulus, internal flaws, and crack depth.

## 3. Results and Discussion

### 3.1. Influential Factors and Interactions in Central Composite Design: RSM Analysis

#### 3.1.1. Slump Flow

**Figure 6:**
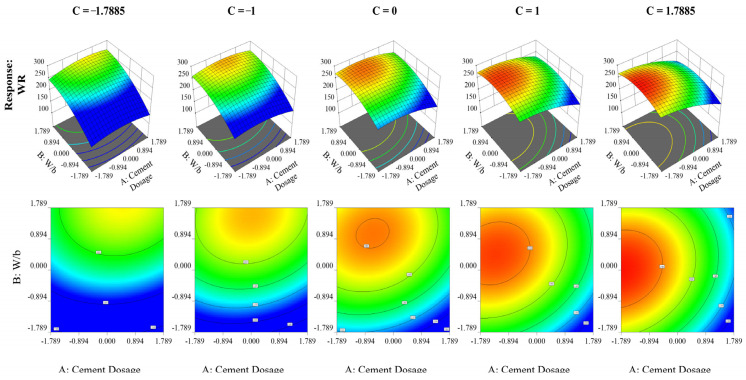
Analysis of factor impact and interactions on RPC slump flow using RSM and contour plots.

Reactive powder concrete (RPC) was at its fresh stage, and the effects of components A, B, and C on the slump flow are shown in Figure 6. The water-to-binder ratio (w/b), which emerged as the most important component influencing this particular reaction, was shown to have a clear linear connection with the slump flow. Table 6 illustrates the workability of the central composite design.

High concentrations of Na_2_O in the GP used in UHPC mixtures (see Table 1) can lead to an alkali–silica reaction [26], which directly affects the compressive strength capacity of the mixture. As a result, it is critical to maintain an even distribution between the dosage of cement (factor A), GP, and superplasticizer (factor C) in order to obtain a flow range between 240 and 260 mm to be considered as a self-compacted mixture according to the European Federation of National Associations Representing for Concrete (EFNARC) [64].

The spread flow value is positively affected, as anticipated, by the presence of factor B (w/b) and the concentration of HRWR (factor C). Among these factors, w/b exerts the most significant influence in the current analysis. However, it should be noted that the high w/b ratio has a detrimental impact on the compressive strength, thus necessitating the consideration of a balanced approach, which will be discussed further below.

Workability increases in direct proportion to the increase in the w/b ratio, with no discernible impact from fluctuations in the percentage of HRWR. When low levels of HRWR are maintained, the concrete has poor workability at all dosage levels. If workability were the only consideration, maintaining values above the center points for the w/b ratio and HRWR would suffice, especially when using a low cement dosage. However, from a financial sense, this strategy may be impractical. As a result, the following part examines the same conditions in order to determine the ideal factor conditions inside the mix while adhering to the previously set limits.

A second-order model was generated using the workability response model, as shown in Table 7. Unlike the negative link between w/b and the amount of HRWR, the interaction between the cement dose and the w/b ratio has no appreciable impact on workability. The w/b ratio has the biggest impact on the straightforward parameters.

#### 3.1.2. Compressive Strength

The average CS at 1, 7, and 28 days are shown in Table 8 after conducting the experiments based on the 18 points of the CCD design. The CS results are analyzed to identify the optimal mixture design in accordance with the conditions stipulated in Table 5.

**Figure 7:**
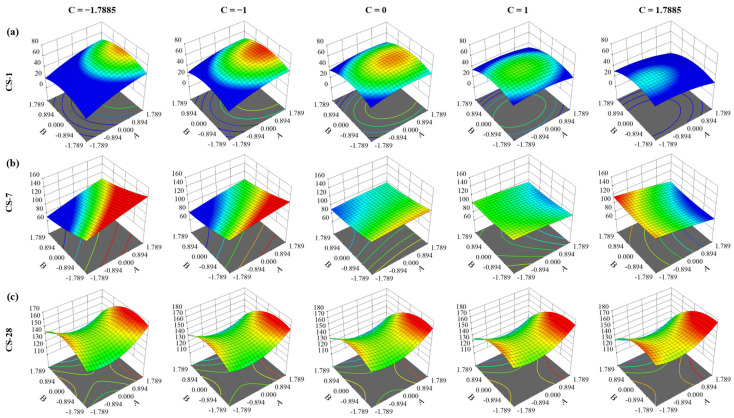
Responses surfaces for impact analysis of factors on RPC’s CS at (**a**) 1 d, (**b**) 7 d, and (**c**) 28 d.

Figure 7 illustrates the impact of factors B and C on the 1-, 7-, and 28-day CS while keeping factor C constant. The results indicate that factor A positively influenced the 1-day CS of RPC, yielding beneficial effects [55,65]. At 1 day, the response surface for CS reveals an inversely proportionate relationship between CS and factors B and C (w/b and superplasticizer, respectively). This is backed by existing research, which shows that a low w/b ratio leads to superior early strength [66]. However, variables B and C exhibited detrimental effects on this response. Furthermore, similar to the 1-day CS, a nonlinear relationship was observed between the 7-day CS and the investigated parameters under consideration.

**Figure 8:**
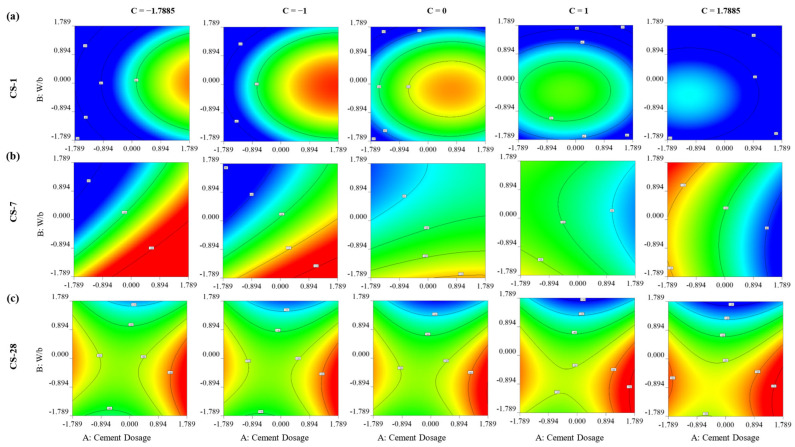
Contour plots for impact analysis of factors on RPC’s CS using at (**a**) 1 d, (**b**) 7 d, and (**c**) 28 d.

According to Wang et al. [67], using polycarboxylate as a superplasticizer promotes the production of C-S-H at later ages, which improves the microstructure of the cementitious matrix at 28 days. However, Puertas et al. [68] stated that the polycarboxylate-based ether HRWR slows silicate hydration (especially the alite phase) and influences ettringite formation, which affects the compressive strength at early ages. This explains the different effects shown by the superplasticizer for the early strengths (1 and 7 days) and for the 28-day strength shown in Figure 7 and Figure 8.

Moreover, too much of this chemical additive (polycarboxylate-based superplasticizer) might make reactive powder concrete (RPC) in its fresh condition stickier than preferred. The maximum compressive strength is attained when factors C and A are both equal to α (2.56 and 673.67 kg/m^3^, respectively) and when factor B is equal to a -α (0.156), according to the response surface for compressive strength at 28 days.

It is worth noting that while the CS varies at 24 h and 7 days, the influence of the interactions is not as pronounced as it is after 28 days. For example, after 28 days, the interaction between water and cement, in addition to being significant, stops to have a proportional effect on resistance and becomes inverse. Given the substantial interaction between cement dose and plasticizer, the latter should be immediately incorporated into the model as a simple factor, as shown in Table 9.

Again, if this were a single answer, it would be appropriate to retain the amount of cement at both its lower and upper limiting values, while keeping w/b at its center point and plasticizer above its center point to maximize the 28-day strength value. Such observations, however, must consider the optimization of all responses, particularly workability and CS28, as explained in the following section.

### 3.2. Simultaneous-Criterion Optimization and Experimental Evaluation

Table 10 depicts the outcomes of the simultaneous-criterion optimization, displaying the expected responses according to the optimized factor levels. The optimized experimental approach resulted in a preferred 29% reduction in OPC dosage and a significant 64% reduction in SF when compared to the normal RPC dosage shown in Table 5. By partially substituting these chemicals with less expensive alternatives such as GP and CaC, the mixture not only reduces its carbon footprint significantly, but also delivers significant long-term cost savings.

### 3.3. Evaluation of Properties of Optimized RPC’s Mixture

Given the global importance of environmental issues, the use of waste materials such as recycled glass in the construction industry has grown in popularity [69,70]. This is especially important in the case of reactive powder concrete (RPC), which requires more cement and hence has a significant carbon footprint impact.

Incorporating locally available admixtures such as calcium carbonate could also assist in overall cost savings in RPC manufacture. However, care must be taken to ensure that the use of mineral admixtures as partial alternatives for ordinary Portland cement (OPC) and silica fume (SF) does not compromise three important features that have a substantial impact on material performance. These characteristics include: (i) achieving proper particle packing density using particle packing theories, (ii) using suitable high-range water-reducing polycarboxylate-based superplasticizers that allow for a low water-to-binder ratio (w/b), and (iii) ensuring proper chemical reactions among components, such as hydraulic and pozzolanic reactions, to generate adequate gel products [49,71].

On the one hand, the presence of GP 28 in the particle size distribution (PSD) curve (Figure 4) helps to maintain the packing density of reactive powder concrete (RPC) while reducing the amount of silica sand and ordinary Portland cement (OPC). Similarly, the addition of calcium carbonate powder with an average particle size of 2 microns can potentially reduce the SF quantity without affecting the packing density much. Furthermore, the use of GP 7, which has a PSD similar to cement, did not reduce RPC’s packing density. On the other hand, optimizing the RPC mixture by introducing GP has a number of upsides. GP has an X-ray diffraction pattern (Figure 3) and a chemical composition (Table 1) that includes a significant amount of non-crystalline silicon oxide. This addition of GP ensures a positive balance of highly pozzolanic components, which helps to strengthen the cementitious paste and allows for a lower SF concentration.

**Figure 9:**
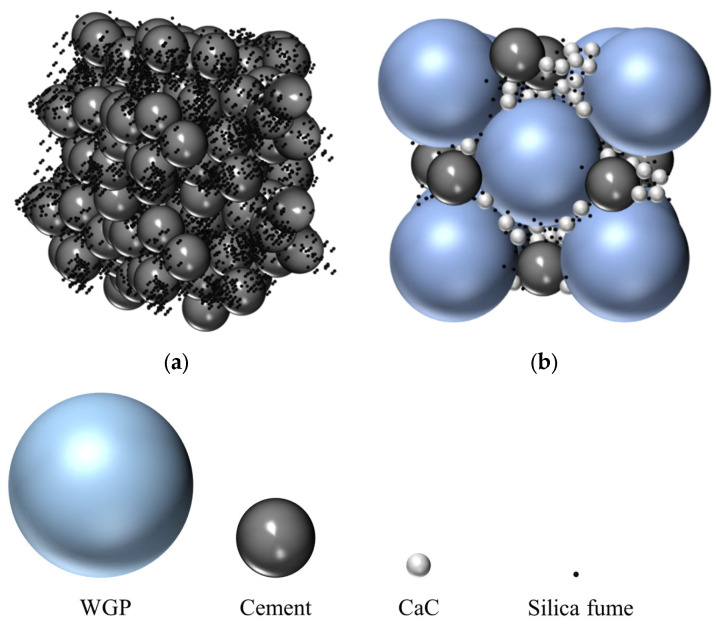
Particle packing density comparison between (**a**) control and (**b**) optimized RPC mixtures.

The w/b ratio and superplasticizer concentration are clearly different between the control RPC dose and the optimized mixture, which is contrasted in detail in Table 10 and Table 11. Additionally, Figure 9 shows the scheme of the paste’s packing density of both RPC mixtures. Due to its alkaline nature and high concentration of Na_2_O (as indicated in Table 1), the use of GP has several advantages, including lowering the amount of water needed and improving flow by dispersing cement particles [65,72,73]. Figure 4d,e show how the nonporous nature of the glass powder particles reduces water absorption, enabling a higher percentage of free water to contribute to the rheological performance of the fresh combination [65].

When SF is present in concrete mixtures in sufficient amounts, it decreases the quantity of free water that is readily accessible, which in turn limits static flow. In order to maintain acceptable workability in mixes with large SF content, greater amounts of superplasticizers are required [65,74,75,76].

Due to the considerable specific surface area of the control sample combination, which is differentiated by its greater SF dose, it requires more water to attain the necessary rheological characteristics [16,46,65,73,77,78]. Additionally, the addition of CaC to the mixture as a partial replacement for ordinary Portland cement (OPC) works as a lubricant.

#### 3.3.1. Compressive Strength

The compressive strength at 90 days in the control mixture was 168.09 MPa ± 4.41, whereas, in the optimized mixture, it measured 161.45 ± 3.38 (3.95% lower), both showing a normal growth of strength (Table 12). Similar results were reported by Ahmad et al. [75], who conducted an optimization study on UHPC utilizing industrial waste materials as a cement replacement and silica fume. They found a compressive strength of 152 MPa at 28 days for UHPC with a flow of 255 mm and up to a 40% reduction in silica fume [69].

The growth rate ratio between the control sample and the optimized mixture decreased over time, reaching a similarity between 28 and 90 days, as depicted in Table 13. The significant variation observed between the ages of 1, 7, and 28 days could be attributed to the higher cement and silica fume content in the control mixture, resulting in greater C-S-H formation at early ages. The increased reactivity of SFs can be attributed to their abundant amorphous silica oxide (Table 1 and Figure 3c), smaller particle size, and larger specific surface area (Figure 4 and Figure 5). The results showed that while the compressive strength values of the optimized RPC were lower than those of the control sample, the performance of the mineral admixtures investigated as SMC of ordinary Portland cement and SF remained within acceptable limits.

#### 3.3.2. Chloride Ion Penetration

**Figure 10:**
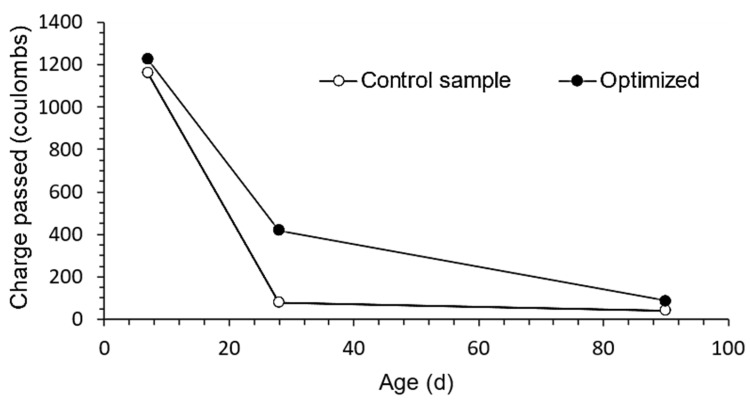
Chloride ion penetration test value mixtures at 7, 28, and 90 days.

At the 28-day mark, there is a noticeable difference between the control and optimized doses (Figure 10), which may be attributed to the SF’s pozzolanic activity and the waste glass’s (starting) operation. Over time, though, these differences become less pronounced.

The improved mixture’s chloride ion penetration was found to be 2.1 times more than that of the control test after 90 days of testing. However, both RPC combination compositions showed insignificant measurements at 28 and 90 days, demonstrating CaC and GP’s outstanding behavior. As a result, it may be inferred that the improved RPC may be used in constructions that must withstand harsh circumstances, such as those prevalent in coastal locations [69,79,80]. At different testing ages, all mixes function exceptionally well overall, with a discernible decrease in permeability to chloride ions with time.

#### 3.3.3. Ultrasonic Pulse Velocity

**Figure 11:**
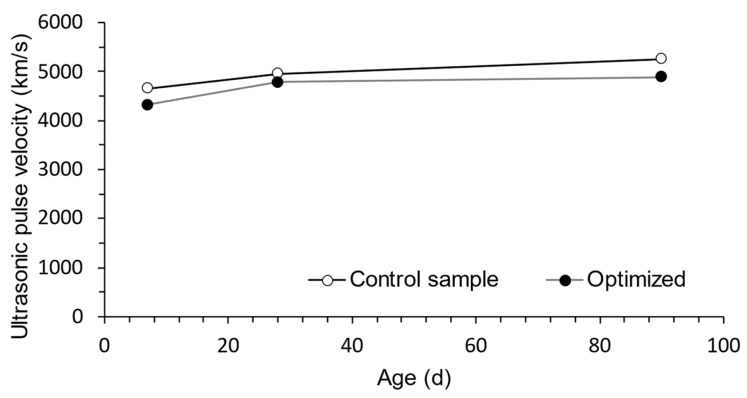
Ultrasonic pulse velocity of mixtures at ages.

A consistent and gradual rise in results is seen over the range of testing ages for ultrasonic pulse velocity analysis. As shown in Figure 11, the optimized RPC mixes follow the control mixture in terms of reaction amplitude. The increased particle packing density inside the concrete matrix, which leads to a higher level of stiffness, can be blamed for this observed tendency. Therefore, the shorter time needed for the ultrasonic wave to pass through the specimen is an indication of the efficient transfer of energy. Notably, for all age stages, the doses of the materials, when ranked according to their respective ultrasonic pulse values, nearly match the dosages ordered by their VPD orders. These results are in line with those found by Nassif et al. [81], who showed the relationship between age progression and an increase in velocity while emphasizing the important roles played by the hydration process and the pozzolanic activity of the cementitious components across the range of UHPC dosages.

#### 3.3.4. Drying Shrinkage

**Figure 12:**
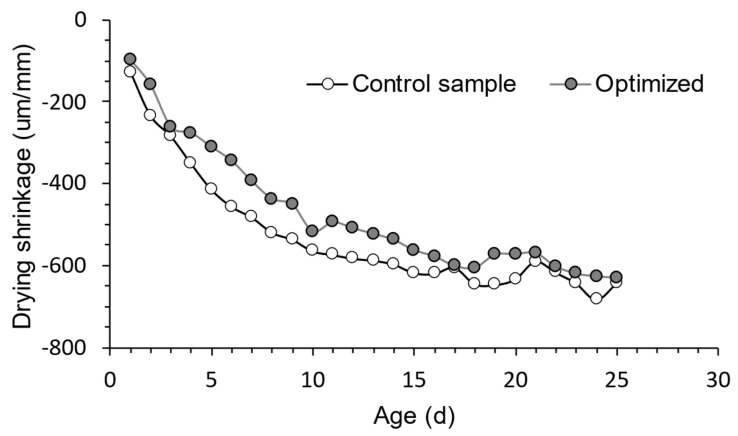
Drying shrinkage of the control sample and optimized RPC mixtures.

According to Figure 12, both the control and optimized RPC combinations showed increased shrinkage rates from days 1 to 15. The shrinkage rate did, however, dramatically decline from day 16 to day 25, which is consistent with findings comparable to those published by other investigators [82,83,84].

Overall, compared to the control test, the improved RPC mixture showed a decreased descent in its shrinkage curve for various measurement ages. This behavior could be attributed to a number of factors, such as slower volumetric changes at young ages, partial replacement of OPC and SF with mineral additives [85], decreased heat of hydration due to lower cement concentration, and reduced water loss during curing as a result of the optimized mixture’s lower water content. As these two components are strongly related in this kind of special concrete, it is important to note that decreasing the amount of SF in the optimum mixture enables a reduction in the amount of water [16,46,65,73,77,78].

Based on the above, it could be inferred that the partial replacement of OPC and SF with mineral additives effectively reduces drying shrinkage, hence lowering the risk of fracture and micro fissure development in the RPC mass.

#### 3.3.5. Cost Savings in the Proposed Optimized RPC

Table 14 provides a detailed financial comparison between the optimized and the traditional reference mixtures. This cost evaluation utilized local market pricing, with the assumption that the expense associated with mixing water is negligible due to its minimal impact on the overall cost. Notably, the traditional control mixture’s expense stood at approximately 435.1 $/m^3^. In contrast, the optimized mixture manifested a cost of just 314.2 $/m^3^, showcasing an appreciable decrease. When quantified, this difference translates to a substantial cost reduction of roughly 27.8% or 120.9 $/m^3^. The data underscores not only the economic advantages inherent in adopting an optimized mixture but also hints at potential enhancements in both its performance and eco-friendliness. The financial ramifications of such a reduction become even more profound when applied to large-scale construction endeavors. This observation, in essence, underscores the pivotal role of continual research and innovation in paving the way for more economically viable and efficient construction materials.

## 4. Conclusions, Implications, and Outlook

A multi-objective simultaneous optimization methodology was adopted in this study to create a financially viable and environmentally sustainable reactive powder concrete (RPC). The strategy included the use of byproducts, such as ground glass powder (GP) as an SCM, with the goal of producing a cost-effective and environmentally friendly concrete that meets many objectives at the same time. The produced material had desirable workability and mechanical properties. The optimized RPC combination from the response surface method was subjected to extensive testing, which included measurements of compressive strength in cylinders, chloride ion penetration, ultrasonic pulse velocity, and drying shrinkage.

The results from this study emphasize the need for overseeing components such as cement dosage, water-to-binder ratio, and superplasticizer content in order to improve RPC’s early and long-term compressive strength while also taking workability and other crucial variables into consideration. The optimized reactive powder concrete (RPC) mixture had a slightly lower compressive strength at 90 days than the control mixture, but both showed normal strength growth over time, with the optimized mixture demonstrating an acceptable performance when mineral admixtures such as cement replacements and silica fume were used. Using powdered granulated blast furnace slag (GP) in ultra-high-performance concrete (UHPC) mixtures can cause an alkali–silica reaction, which has a direct effect on the mixture’s capacity for compressive strength. In influencing the slump flow of reactive powder concrete (RPC) at its fresh stage, the water-to-binder ratio (w/b) is a critical factor. It displays a distinct linear relationship with the slump flow, demonstrating its major impact on workability.

The use of superplasticizers based on polycarboxylates stimulates the formation of calcium-silicate-hydrate (C-S-H), which improves the microstructure of the cementitious matrix. It also has an effect on silicate hydration and ettringite production. However, too much of this chemical addition might cause stickiness in fresh RPC.

When compared to the control mixture, the optimized reactive powder concrete (RPC) dosage has a lower water-to-binder ratio and superplasticizer concentration, which is influenced by factors such as silica fume-specific surface area, the reduced presence of free water, the lubricating effect of calcium carbonate (CaC), and the water-reducing and dispersing properties of glass powder, resulting in improved rheological performance.

The pozzolanic activity of silica fume and waste glass resulted in noticeable differences between the optimized reactive powder concrete (RPC) mixture and the control mixture at the 28-day mark; however, both mixtures demonstrated satisfactory performance over time, with improved chloride ion penetration resistance and reduced permeability to chloride ions, making it suitable for construction in harsh environments such as coastal areas.

The optimized reactive powder concrete (RPC) with partial substitution of OPC and SF by mineral admixtures showed less drying shrinkage than the control RPC dosage, resulting in slower volumetric change at early ages and a lower likelihood of the formation of cracks and microcracks, highlighting the effectiveness of mineral admixtures in minimizing essential drying shrinkage and improving RPC’s mass integrity.

To summarize, as the inclusion of SCMs in the RPC mixture can influence the hydration kinetics and microstructure of the material, future research should strive to understand the underlying chemical mechanisms, hydration products, and pore structure evolution of blended RPC in order to improve its overall performance and potential considering the previously identified limitations and constraints. This analysis would play a role in ensuring a balanced strategy that meets both workability and financial goals.

In the current phase of our investigation, we have focused on an RPC formulation that uniquely incorporates waste glass and micro calcium carbonate. While our assessments have been confined to laboratory settings, the initial findings, including notable workability and compressive strength, hint at its viability for real-world engineering scenarios. This RPC not only offers an eco-friendly construction alternative by repurposing waste, but also presents potential economic efficiencies by substituting pricier traditional elements. Furthermore, its robust performance metrics, such as resistance to chloride infiltration, suggest broad applicability in diverse construction contexts, marking a significant stride in sustainable infrastructure development.

Future research could further explore alternative materials and methodologies to enhance the properties of RPC beyond those of traditional concrete. Exploring the incorporation of nanomaterials, diverse fiber types, or supplementary cementitious materials offers promising avenues. Furthermore, refining mixing protocols, curing processes, and particle packing strategies may lead to significant advancements. 

## Figures and Tables

**Table 1:** Chemical characterization of granular RPC-making raw materials.

Oxides (%)	Cement	SF	CaC	GP 28
SiO_2_	20.01	94.01	0.91	72.48
CaO	63.41	3.71	55.73	9.51
Na_2_O	0.14	0.21	0.01	11.61
Al_2_O_3_	5.62	0.62	0.2	1.43
SO_3_	2.59	0.05	0.14	0.02
Fe_2_O_3_	3.02	0.22	0.06	0.62
Others	1.92	0.59	0.79	2.38

**Table 2:** Physical characterization of granular RPC-making raw materials.

Properties	Cement	SF	CaC	GP 28
L.O.I (%)	3.29	0.59	42.12	2.22
Specific gravity	3.14	2.21	2.74	2.61
d50 (μm)	9	0.15	2	28

**Table 3:** The RPC mixing proportions for the design points (DP) of the CCD.

Mix	Coordinates (A, B, C)	Cement	SF	WGP	Calcium Carbonate	Sand	Water	Superplasticizer	w/b
1	(−1, −1, −1)	590	100	328	231	884	200	22	0.16
2	(1, −1, −1)	650	100	296	202	895	200	22	0.16
3	(−1, 1, −1)	590	100	328	231	851	212	22	0.17
4	(1, 1, −1)	650	100	296	202	861	212	22	0.17
5	(−1, −1, 1)	590	100	328	231	881	200	26	0.16
6	(1, −1, 1)	650	100	296	202	892	200	26	0.16
7	(−1, 1, 1)	590	100	328	231	849	212	26	0.17
8	(1, 1, 1)	650	100	296	202	859	212	26	0.17
9	(0, 0, 0)	620	100	320	223	851	208	24	0.165
10	(0, 0, 0)	620	100	320	223	851	208	24	0.165
11	(−α, 0, 0)	566	100	341	244	859	206	24	0.165
12	(α, 0, 0)	674	100	282	190	885	206	24	0.165
13	(0, −α, 0)	620	100	320	223	881	197	24	0.156
14	(0, α, 0)	620	100	320	223	821	220	24	0.174
15	(0, 0, −α)	620	100	320	223	854	208	20	0.165
16	(0, 0, α)	620	100	320	223	849	208	28	0.165
17	(0, 0, 0)	620	100	320	223	851	208	24	0.165
18	(0, 0, 0)	620	100	320	223	851	208	24	0.165

**Table 4:** Factor values for each of the coordinates in the CCD.

Factor	Units	Factor’s Coordinates in the CCD				
		−α	−1	0	1	α
A	kg/m^3^	566.33	590	620	650	673.67
B	-	0.156	0.16	0.165	0.17	0.174
C	(%, vol.)	1.84	2	2.2	2.4	2.56

**Table 5:** Values of factor’s coordinates in the CCD.

Name	Goal	L	U
A: Cement dosage	Minimize	-	-
B: W/b	Range	0.14	0.18
C: HRWR concentration	Minimize	-	-
CS28	Maximize	-	-
WR	Range	240	260

**Table 6:** Workability for mixes.

Mix	Workability (mm)
M1	223.50 ± 4.12
M2	206.50 ± 2.52
M3	265.50 ± 0.58
M4	263.00 ± 1.83
M5	272.50 ± 2.89
M6	239.50 ± 0.58
M7	291.00 ± 0.00
M8	258.50 ± 1.00
M9	264.50 ± 2.08
M10	267.00 ± 3.46
M11	263.50 ± 1.29
M12	241.50 ± 2.89
M13	211.50 ± 0.58
M14	276.50 ± 0.58
M15	245.50 ± 0.58
M16	263.50 ± 0.58
M17	273.00 ± 0.00
M18	272.25 ± 1.89

**Table 7:** Regression coefficients and *p*-values for the workability response model.

Time	Intercept	A	B	C	AB ^(^*^)^	AC ^(^**^)^	BC	A^2^	B^2^	C^2^
WR	269.286	−8.636	17.519	9.389	1.875	−5.750	−7.625	−5.055	−7.712	−4.430
*p*-value	-	0.0028	<1 × 10^−4^	0.0018	0.490	0.0606	0.0210	0.0307	0.0045	0.0499

^(^*^)^ One factor or interactions *p*-value > 0.1. The coefficient is not included in the regression model. ^(^**^)^ One factor or interactions 0.1 ≤ *p*-value ≥ 0.05. The coefficient is included in the regression model.

**Table 8:** Development of CS of UHPC with different proportions.

Mix	Compressive Strength (MPa)		
	1 d	7 d	28 d
M1	44.33 ± 8.46	103.48 ± 5.90	147.40 ± 8.21
M2	54.49 ± 3.81	114.84 ± 6.15	156.06 ± 11.90
M3	43.11 ± 8.23	88.73 ± 7.22	142.24 ± 7.65
M4	53.86 ± 1.08	100.09 ± 4.36	142.39 ± 8.89
M5	49.55 ± 9.08	104.02 ± 17.77	152.06 ± 4.53
M6	47.40 ± 2.64	96.62 ± 4.53	160.24 ± 8.32
M7	45.62 ± 4.18	101.58 ± 10.61	139.50 ± 2.79
M8	43.46 ± 7.83	96.36 ± 6.68	139.90 ± 9.44
M9	60.14 ± 4.21	99.42 ± 6.98	147.19 ± 2.33
M10	56.52 ± 5.98	99.42 ± 9.09	152.00 ± 10.60
M11	48.89 ± 5.44	94.30 ± 10.50	157.28 ± 2.58
M12	57.24 ± 6.01	100.94 ± 15.54	164.74 ± 1.66
M13	48.55 ± 7.81	109.95 ± 16.42	144.10 ± 5.00
M14	44.83 ± 3.23	94.58 ± 10.23	119.41 ± 3.88
M15	49.82 ± 6.53	103.89 ± 7.49	148.51 ± 4.48
M16	46.44 ± 2.59	99.09 ± 12.02	148.57 ± 6.45
M17	54.57 ± 10.00	98.03 ± 6.15	142.88 ± 3.78
M18	56.35 ± 6.40	98.57 ± 5.35	146.09 ± 7.66

**Table 9:** Regression coefficients and *p*-values for the compression strength response model at 1, 7, and 28 days.

	Intercept	A	B	C	AB	AC	BC	A^2^	B^2^	C^2^
CS-1	56.833	2.189	−1.137	−1.097 ^(^**^)^	0.071 ^(^*^)^	−3.152	−0.752 ^(^**^)^	−1.527	−3.517	−3.069
*p*-value		0.0043	0.0681	0.0762	0.9227	0.0030	0.3235	0.0210	2 × 10^−4^	6 × 10^−4^
CS-7	98.849	1.527	−4.146	−1.191	0.271 ^(^*^)^	−4.418	3.350	−0.330 ^(^**^)^	1.123	0.880
*p*-value		<1 × 10^−4^	<1 × 10^−4^	2 × 10^−4^	0.2756	<1 × 10^−4^	<1 × 10^−4^	0.0884	3 × 10^−4^	0.0011
CS-28	146.981	2.134	−6.659	0.256 ^(^**^)^	−2.036 ^(^**^)^	−0.031 ^(^*^)^	−1.759 ^(^**^)^	4.426	−4.716	0.529 ^(^*^)^
*p*-value		0.0087	<1 × 10^−4^	0.6781	0.0375	0.9696	0.0625	1 × 10^−4^	<1 × 10^−4^	0.3904

^(^*^)^ One factor or interactions *p*-value > 0.1. The coefficient is not included in the regression model. ^(^**^)^ One factor or interactions 0.1 ≤ *p*-value ≥ 0.05. The coefficient is included in the regression model.

**Table 10:** Optimized RPC mixture: factors and responses.

Optimized RPC Coordinates			Factor’s Values			Model’s Responses	
A	B	C	A (Cement in kg/m^3^)	B (w/b)	C (%sp)	Slump (mm)	R28 (MPa)
−0.894	0	−0.745	603	0.165	2	250	155

**Table 11:** RPC mixture proportions in kg/m^3^.

Material	Cement	SF	Waste Glass	Calcium Carbonate	Sand	Superplasticizer	Water
Control	852	272	0	0	834	26.5	245
Optimized	590	100	335	257	778	21.5	211

**Table 12:** Variation of CS of control and optimized RPC mixtures at 1, 7, 18, and 90 days.

Mixtures	1 d	7 d	28 d	90 d
Control sample	74.33 ± 2.11	123.48 ± 2.95	167.06 ± 2.85	168.09 ± 4.41
Optimized	48.55 ± 2.88	103.25 ± 3.65	156.27 ± 4.14	161.45 ± 3.38

**Table 13:** CS growth rate of mixtures.

Mixtures	1 to 7 d	7 to 28 d	28 to 90 d
Control sample	66.1%	35.3%	0.61%
Optimized	112.7%	51.3%	3.31%

**Table 14:** Cost analysis for the optimized and reference mixtures.

Material	Cement	SF	Waste Glass	Calcium Carbonate	Sand	Superplasticizer	Total ($/m^3^)
Cost ($/kg)	0.114	0.566	0.026	0.050	0.054	3.553	–––
Control	97.53157895	153.8947368	0	0	44.99210526	94.14473684	435.1
Optimizad	67.53947368	56.57894737	8.815789474	12.85	41.97105263	76.38157895	314.2

## Data Availability

Not applicable.
